# Carbon Monoxide Induced Metabolic Shift in the Carboxydotrophic *Parageobacillus thermoglucosidasius* DSM 6285

**DOI:** 10.3390/microorganisms9051090

**Published:** 2021-05-19

**Authors:** Habibu Aliyu, Ronnie Kastner, Pieter de Maayer, Anke Neumann

**Affiliations:** 1 Section II: Technical Biology, Institute of Process Engineering in Life Science, Karlsruhe Institute of Technology, 76131 Karlsruhe, Germany; ronnie-kastner@web.de; 2 School of Molecular & Cell Biology, Faculty of Science, University of the Witwatersrand, Johannesburg 2000, South Africa; pieter.demaayer@wits.ac.za

**Keywords:** acetate, biological water gas shift reaction, carbon monoxide, lactate, *Parageobacillus thermoglucosidasius*, reducing equivalents

## Abstract

*Parageobacillus thermoglucosidasius* is known to catalyse the biological water gas shift (WGS) reaction, a pathway that serves as a source of alternative energy and carbon to a wide variety of bacteria. Despite increasing interest in this bacterium due to its ability to produce biological hydrogen through carbon monoxide (CO) oxidation, there are no data on the effect of toxic CO gas on its physiology. Due to its general requirement of O_2_, the organism is often grown aerobically to generate biomass. Here, we show that carbon monoxide (CO) induces metabolic changes linked to distortion of redox balance, evidenced by increased accumulation of organic acids such as acetate and lactate. This suggests that *P. thermoglucosidasius* survives by expressing several alternative pathways, including conversion of pyruvate to lactate, which balances reducing equivalents (oxidation of NADH to NAD^+^), and acetyl-CoA to acetate, which directly generates energy, while CO is binding terminal oxidases. The data also revealed clearly that *P. thermoglucosidasius* gained energy and grew during the WGS reaction. Combined, the data provide critical information essential for further development of the biotechnological potential of *P. thermoglucosidasius*.

## 1. Introduction

*Parageobacillus thermoglucosidasius* [1,2] is a facultatively anaerobic thermophile isolated from diverse habitats, including soil, hot springs and plants [3]. This species is metabolically versatile and capable of utilizing a broad range of carbohydrates, including sugar monomers such as glucose and xylose [3,4,5]. The ability of *P. thermoglucosidasius* to produce thermostable enzymes, its growth temperature range and metabolic versatility led to an increased interest in its exploration for various biotechnological applications [6,7].

Previous studies have elucidated in detail the central carbon metabolism of *P. thermoglucosidasius* based on ^13^C-based flux [8], and qPCR and proteomics [9] under aerobic and microaerobic growth on glucose. When the bacterium was cultured on glucose, the carbon flux was channelled through the TCA cycle and oxidative pentose phosphate pathway under high redox and through organic acids and ethanol under microaerobic conditions [8,9]. Although both studies demonstrated the glyoxylate shunt activity in *P. thermoglucosidasius*, a ^13^C-based flux analysis [8] failed to resolve the carbon flux through this pathway under the conditions tested. However, Loftie-Eaton et al. [9] reported down-regulation of the glyoxylate shunt under microaerobic conditions based on the expression profiles of genes and proteins associated with the pathway.

*P. thermoglucosidasius* produces biohydrogen (H_2_) via the water gas shift (WGS) reaction pathway (CO  +  H_2_O → CO_2_  +  H_2_ ΔG^Oʹ^ = −20 kJ/mol CO) [10]. It utilises a CO dehydrogenase (CODH)/hydrogen-evolving hydrogenase (HEH; Phc in *P. thermoglucosidasius*) complex to oxidise carbon monoxide (CO) to CO_2_ plus electrons which are used for reducing the proton (H^+^) from H_2_O to H_2_ [11,12,13,14]. Thus far, no empirical evidence exists regarding whether this group 4a hydrogenase, harboured by *P. thermoglucosidasius* [15], translocates protons through the cellular membrane to generate energy. However, an ATP-yielding transmembrane proton gradient was demonstrated in the purple non-sulphur photosynthetic bacterium *Rubrivivax gelatinosus* during the WGS reaction, suggesting that hydrogenase couples reduction of proton to proton translocation [16]. Several other members of group 4 hydrogen-evolving hydrogenases have been reported to conserve energy by translocating protons and sodium ions [13,17,18].

The phylogenetic and physiological diversity of hydrogenogenic carboxydotrophs was recently reviewed by Fukuyama et al. [19]. Diverse CO-oxidizing taxa in the phyla *Firmicutes* and *Proteobacteria*, as well as Archaea have been described with various temperature adaptations, including thermophilic and mesophilic forms [19,20]. However, most of the reported CO oxidizers belong to the phylum *Firmicutes*, which accounts for about 50% of the known diversity and comprises mainly strict anaerobes [14,21,22]. Of the various phyla potentially capable of anaerobic CO oxidation, two groups of facultative anaerobes are of particular interest because of their potential application in oxygen-tolerant hydrogen production systems. The first group includes marine mesophilic *Proteobacteria* such as *Ferrimonas futtsuensis* DSM18154 [21] and *Photobacterium marinum* AK15 [22], whose potential for hydrogen production is based only on genomic analyses [23]. Conversely, CO oxidation and hydrogen production via the WGS reaction have been demonstrated in the facultatively anaerobic *P. thermoglucosidasius* [14,24]. This organism grows aerobically until O_2_ depletion, whereafter there is a lag phase during which biomass decreases. If CO is supplied, H_2_ is produced with a concomitant increase in biomass [25].

*P. thermoglucosidasius* and the related *Geobacillus thermodenitrificans* DSM 465^T^ and *P. toebii* DSM 14590^T^ exhibit varying degrees of tolerance to CO at a 50% CO: 50% air ratio [10]. However, the latter two strains produced relatively less biomass (based on OD_600_), suggesting less tolerance relative to *P. thermoglucosidasius*. Inferences from these comparisons are, however, limited due to the lack of appropriate controls without CO. Despite the assumption of CO tolerance, previous studies revealed that at higher CO concentration, the growth of *P. thermoglucosidasius* was greatly impaired [25].

In spite of its ability to oxidise CO, to date, initial growth of the organism to sufficient biomass is only achievable by growing it aerobically and providing organic substrates (e.g., glucose), resulting in an extended lag-phase before the start of the WGS reaction [25]. Therefore, further strategies for improving the WGS reaction in *P. thermoglucosidasius*, whether via optimizing growth parameters or genetic manipulations, require the understanding of the physiological consequences of growth in the presence of CO plus organic substrates. Here, physiological changes induced by CO and its implication on growth and energetics of the carboxydotroph *P. thermoglucosidasius* DSM 6285 were evaluated in bioassays combined with GC and HPLC analyses of the gases and selected metabolites. To the best of our knowledge, no previous studies on the effects of CO on the metabolism of *P. thermoglucosidasius* have been published.

## 2. Materials and Methods

### 2.1. Strain, Media and Growth Condition

*P. thermoglucosidasius* DSM 6285, obtained from DSMZ Germany, is maintained as a glycerol stock stored in a freezer at −80 °C. The strain was revived in a modified Luria–Bertani (mLB) medium comprising 10 g/L tryptone, 5 g/L yeast extract, 5 g/L NaCl, 1.25 mL/L of 10 g/L NaOH and 1 mL/L of each of the filter-sterilized stock solutions nitrilotriacetic acid (1.05 M), MgSO_4_·7H_2_O (0.59 M), CaCl_2_·2H_2_O (0.91 M) and FeSO_4_·7H_2_O (0.04 M). A pre-culture was grown for 14 h in a 100 mL shake flask containing 20 mL mLB medium inoculated with 10 µL of the glycerol stock and incubated at 60 °C and 120 rpm in Infors Thermotron, Switzerland. Cultivations were conducted in 250 mL serum bottles sealed with a rubber stopper (top: 18 mm, bottom 14 mm and height 20 mm, Rotilabo ^®^, Carl Roth, Karlsruhe, Germany). The bottle contains the ASM medium as previously described [24,25] with the following modifications. The ASM medium was supplemented per litre with 1 g glucose, 0.02 mM biotin (Carl Roth, Karlsruhe, Germany), 20 mL MEM amino acids solution (50×), 10 mL MEM non-essential amino acids solution (100×) and 10 mL MEM vitamin solution (100x; Thermo Scientific, Schwerte, Germany).

The headspace in each serum bottle was initially reconstituted to 100% N_2_ atmosphere. To test the effects of carbon monoxide (CO) on growth of the bacterium, the initial CO composition in the headspace was set at ~0:100, 25:75, 50:50, 75:25, 100:0 CO to N_2_ ratios. Due to the O_2_ requirement for the initial growth of *P. thermoglucosidasius* [6], ~1.3 to 1.4 mmol (40 mL at 1 bar pressure) of O_2_ was injected in each bottle prior to the start of the experiments. The pre-culture was used to inoculate the bottles at an initial absorbance (OD_600_) of 0.1. All cultures were grown at 60 °C on a rotary shaker at 120 rpm in triplicate (representing three distinct biological replicates) for a duration of 78 h. One endpoint sample was collected after 152 h.

### 2.2. Sampling and Analytics

Approximately, 3 mL of the headspace was sampled at each time point and analysed using a 300 Micro GC gas analyser (Inficon, Switzerland) connected with Molsieve columns and PLOT Q for data acquisition). Calculation of the gas composition was based on the ideal gas law as previously described [24,25]. The pressure was determined before and after the gas measurements using a manometer (GDH 14 AN, Greisinger electronic, Regenstauf, Germany). A liquid sample (1 mL) was collected at each sampling time for growth and subsequent HPCL analysis. The absorbance (OD_600_) and pH were measured using the Ultrospec 1100 pro spectrophotometer (Amersham Biosciences, Uppsala, Sweden) and Profilab pH 597 (Xylem Analytics, Weilheim, Germany).

Seven end-products of central carbon metabolism, namely, acetate, ethanol, formate, fumarate, glyoxylate, lactate, and succinate were monitored over 0.1–1.0 g/L concentration ranges using HPLC. Thawed supernatant (previously stored at −20 °C) of selected samples were filtered using a nylon filter (0.22 µm, Carl Roth, Karlsruhe, Germany) and 100 μL of each was dispensed in 1.5 mL HPLC autosampler vials fitted with micro-inserts. The samples were analysed using an Agilent 1100 series HPLC system (Agilent Technologies, Waldbronn, Germany) connected to a wavelength detector and refractive index detector with a 50 mm long pre-column (model Rezex ROA-Organic Acid H+ (8%) Guard Column) and a 300 mm long separation column (model Rezex ROA-Organic Acid H+ (8%). Analyses were performed using 5 mM H_2_SO_4_ mobile phase, 50 °C column temperature, 0.5 mL/min flow rate for 40 min per sample and injection volume of 10 μL. Chemstation (Agilent Technologies) was used for data acquisition and data handling.

All datasets were summarised using Microsoft Excel and figures plotted using OriginPro 2021 9.8.0.200 (Academic).

## 3. Results

### 3.1. Carbon Monoxide Enhances Growth of P. thermoglucosidasius DSM 6285

**Figure 1:**
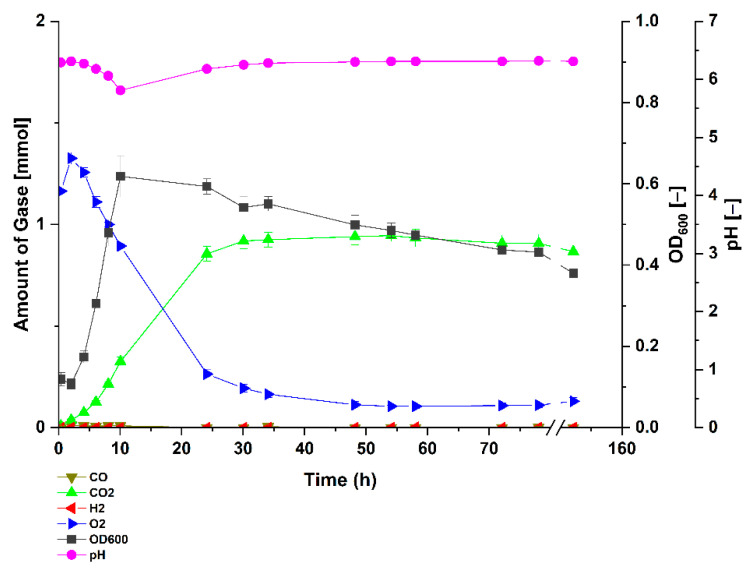
Gas composition and growth of *P. thermoglucosidasius* DSM 6285 cultivated under 0% carbon monoxide atmosphere. The strain was grown in stoppered 250 mL serum bottles containing 50 mL of modified ASM medium and an initial O_2_ concentration of ~1.2 to 1.4 mmol (40 mL at 1 bar pressure). Reported values represent means of triplicate experiments ± standard deviation.

**Figure 2:**
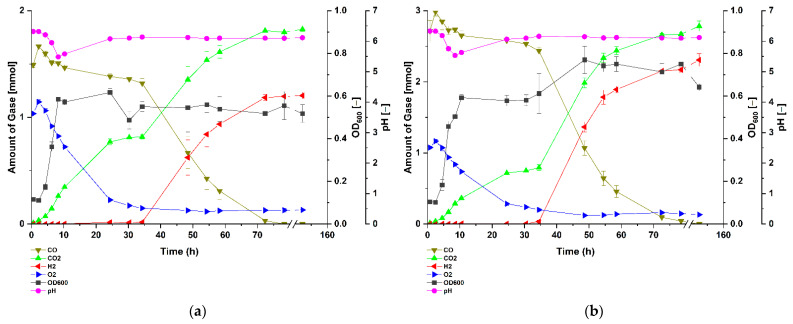
Gas composition and growth of *P. thermoglucosidasius* DSM 6285 cultivated under (**a**) ~25% and (**b**) ~50% carbon monoxide atmosphere. The strain was grown in stoppered 250 mL serum bottles containing 50 mL of modified ASM medium and an initial O_2_ concentration of ~1.2 to 1.4 mmol (40 mL at 1 bar pressure). Reported values represent means of triplicate experiments ± standard deviation.

To examine the effects of carbon monoxide on growth and biological WGS reaction, *Parageobacillus thermoglucosidasius* DSM 6285 was exposed to atmospheres containing different concentrations of CO (~0, 25, 50, 75 and 100%) in serum bottles. Because the bacterium is unculturable directly on CO as single carbon source, the media and headspace contained 1 g/L glucose and additionally, ~1.2 to 1.4 mmol of O_2_, respectively. Cultivation of *P. thermoglucosidasius* in an atmosphere devoid of CO showed that the strain grew rapidly to a maximum absorbance (OD_600_) of 0.62 after ~10 h (Figure 1), which corresponded to the point at which the lowest pH value of 5.81 was observed. This was followed by a rapid decline in the absorbance over the rest of the experiment as the O_2_ level dropped to a minimum value of 0.11 mmol. Since no CO was added, no H_2_ was detected under this condition. By contrast, cultivation of the strain in an atmosphere containing 25, 50, 75 or 100% of CO showed varying patterns of growth, differences in the duration of the WGS reaction lag-phase and H_2_ yield. Under ~25% CO atmosphere, the strain showed a similar growth pattern as the 0% CO cultures (Figure 1) within the first few hours of cultivation (Figure 2a), reaching an OD_600_ value of 0.58 at ~8.29 h. Unlike in the ~0% CO cultures (Figure 1), the absorbance for the strain growing under ~25% CO showed a marginal decrease before reaching a similar maximum OD_600_ value of 0.62 around 24.11 h post-inoculation. Prior to the commencement of the WGS reaction, the absorbance dropped and then increased to a stable value until the end of the experiment, suggesting that the presence of CO influenced the growth of the organism during the WGS reaction lag-phase.

**Figure 3:**
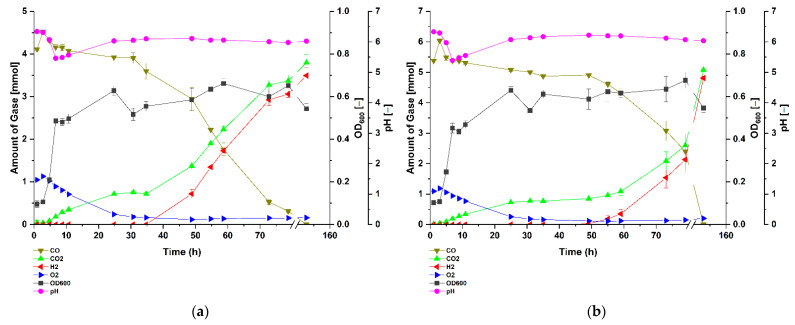
Gas composition and growth of *P. thermoglucosidasius* DSM 6285 cultivated under (**a**) ~75% and (**b**) ~100% carbon monoxide atmosphere. The strain was grown in stoppered 250 mL serum bottles containing 50 mL of modified ASM medium and an initial O2 concentration of ~1.2 to 1.4 mmol (40 mL at 1 bar pressure). Reported values represent means of triplicate experiments ± standard deviation.

Cultivation of the strain under ~50% CO provides greater contrast regarding the effect of CO on the WGS reaction lag-phase (Figure 2b). The absorbance values suggest the presence of multiple growth phases involving initial rapid increase to an OD_600_ value of 0.50 around 8.50 h post-inoculation, corresponding to the point at which the lowest pH value of 5.53 was observed. The next phase occurred when the pH of the medium increased again and the OD_600_ value increased to 0.59 around 10.50 h. Notably, the absorbance stabilized until WGS started and then reached a maximum OD_600_ value of 0.78 around 48 h. The absorbance only declined with the complete depletion of CO, suggesting that the bacterium gained energy and possibly carbon for growth via the WGS reaction. The growth of the bacterium at ~75 and 100% CO (Figure 3) atmospheres shares the general trend observed for the ~50% CO cultures with the exception that in both treatments the observed OD_600_ values were consistently lower than those observed in the ~50% CO cultures during the WGS reaction phase. A notable difference was that, at ~100% CO atmosphere, there was a pronounced extension in WGS reaction lag-phase, which started at around 59 h post-inoculation (Figure 3b), suggesting the occurrence of critical CO-induced metabolic changes during this stage of growth. By contrast, the duration of this lag-phase was ~34 h for each of the ~25, 50 and 75% CO cultures (Figure 2a,b and Figure 3a). Despite these differences, the bacteria completely utilized the various amounts of CO in the headspace to produce H_2_ and CO_2_ (Figure 2a,b and Figure 3a,b).

### 3.2. P. thermoglucosidasius DSM 6285 Cope with Carbon Monoxide by Rerouting Metabolic Intermediates

**Figure 4:**
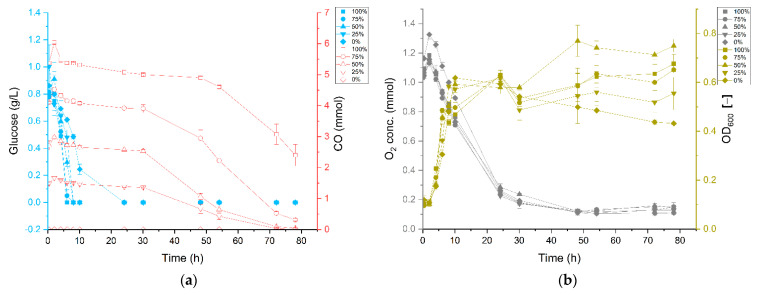
Growth of *P. thermoglucosidasius* DSM 6285 under different initial carbon monoxide atmospheres. (**a**) Concentrations of carbon monoxide (0, 25, 50, 75 and 100% CO) and glucose and (**b**) the corresponding OD_600_ and O_2_ concentrations over the duration of cultivation. Reported values represent means of triplicate experiments ± standard deviation. CO, glucose, OD_600_ and O_2_ are shown in red, blue, dark yellow and grey colours, respectively.

To gain further insight into the observed influence of CO on the growth of *P. thermoglucosidasius* DSM 6285, the supernatants collected over the course of the cultivation were examined for various metabolites. For cultures exposed to an atmosphere comprising ~25, 50, 75 and 100% CO, the glucose (used as organic carbon source) was completely utilized around 10 h post-inoculation, with minor differences observed among measurements for these conditions (Figure 4a). Conversely, ~25% of the glucose was present at the same time (around 10 h) in cultures growing without CO, suggesting that CO enhanced the utilization of glucose. However, the detected difference in glucose concentration is not immediately apparent from the absorbance data as the 0% CO cultures show similar OD_600_ values to those of the CO-treated cultures (Figure 4b). Similarly, the trend in O_2_ concentration, which provides a measure of aerobic respiration, did not vary greatly among the treatments.

**Figure 5:**
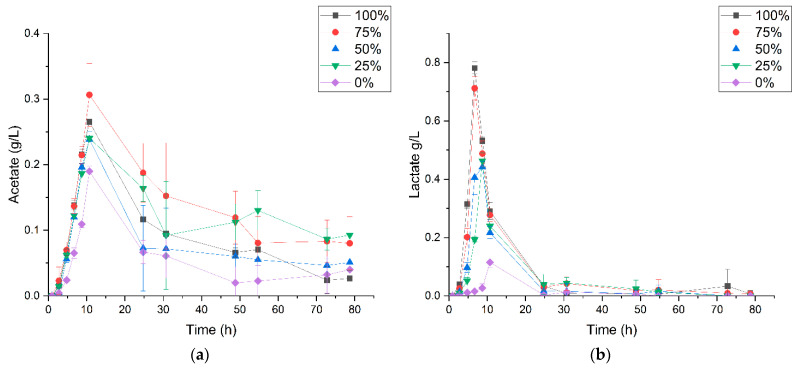
Concentrations of (**a**) acetate and (**b**) lactate during growth of *P. thermoglucosidasius* DSM 6285 under different initial carbon monoxide atmospheres (0, 25, 50, 75 and 100% CO). Maximum concentrations of both compounds occurred at ~10 h when ~50% of the initial O_2_ was depleted. Reported values represent means of triplicate experiments ± standard deviation.

**Figure 6:**
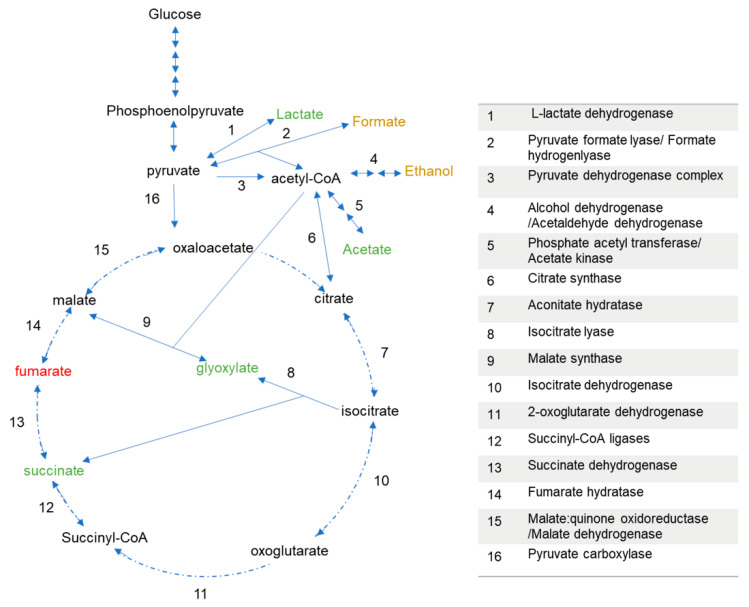
Glucose metabolism pathway and putative enzymes predicted/or shown to occur in *P. thermoglucosidasius* [26,27]. Green (detected above 0.1 g/L), orange (detected below 0.1 g/L) and red (not detected) fonts indicate substances analysed in the current work and black fonts indicate substance not analysed. Multiple arrows show pathways with two or more reactions.

Metabolite analysis further revealed higher concentrations of acetate ranging between 0.24 to 0.31 g/L in CO-treated cultures compared to 0.18 g/L in cultures without CO (Figure 5a). Additionally, the data suggest that the presence of CO induces a more rapid production of acetate in the former cultures regardless of the CO concentration. Similarly, there were higher concentrations of lactate in CO-grown cultures relative to the 0% CO cultures (Figure 5b). A maximum of 0.12 g/L of lactate was produced at ~11-h post-inoculation in the cultures without CO compared to a maximum range of 0.46–0.78 g/L in the CO-treated bottles. In contrast to acetate, CO induction of lactate production evidently depends on the CO concentration. For instance, at ~7 h post-inoculation, the concentration of lactate was at the maximum value of 0.78 g/L in ~100% CO cultures, compared to 0.71, 0.41 and 0.19 for cultures grown under 75, 50 and ~25% CO atmosphere, respectively. Combined, the increased production of acetate and lactate suggests that CO induces the rerouting of metabolites, likely from pyruvate to organic acids (Figure 6; to be discussed further). It could be deduced that the extended duration of the WGS reaction lag-phase in the ~100% CO-grown cultures may be connected to the accumulation of these acids at higher concentrations. Remarkably, prior to the start of CO oxidation, lactate was completely consumed in all cultures irrespective of the CO concentration. In contrast, some amount of acetate persisted throughout the cultivation period.

**Figure 7:**
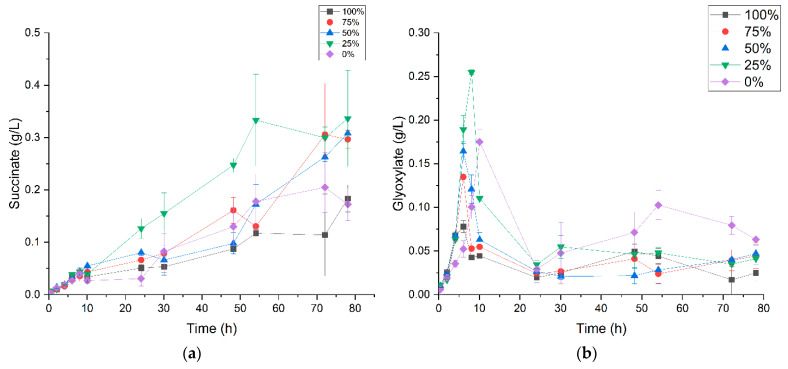
Concentrations of (**a**) succinate and (**b**) glyoxylate during growth of *P. thermoglucosidasius* DSM 6285 under different initial carbon monoxide atmospheres (0, 25, 50, 75 and 100% CO). Glyoxylate reached maximum concentration at ~10 h when ~50% of the initial O_2_ was consumed while succinate accumulates during the cultivation. Reported values represent means of triplicate experiments ± standard deviation.

HPLC metabolite quantification also revealed that in addition to the above-mentioned organic acids, *P. thermoglucosidasius* DSM 6285 produced succinate (Figure 7a), presumably via the glyoxylate shunt (Figure 6), evident by the corresponding turnover of glyoxylate (Figure 7b). The concentrations of succinate ranged between a maximum of 0.34 g/L and 0.17 g/L in ~25% CO and 0% CO conditions, respectively, and increased progressively over the course of the cultivation. The HPLC analysis did not detect fumarate, indicating that succinate accumulated as a metabolic end-product. In contrast, the amounts of glyoxylate, which reached a peak range of 0.08–0.26 g/L between 6- and 10-h, dropped to values below 0.05 g/L after 24 h in all cultures, suggesting removal via malate synthesis. The current analysis, however, did not include malate. The concentration of glyoxylate decreases with increases in CO concentration for all CO-treated cultures. However, the ~25% CO-treated culture gave a higher maximum glyoxylate concentration of 0.26 g/L than that of the 0% CO control (0.18 g/L). Two other compounds, ethanol and formate had maximum concentrations (<0.1 g/L) bordering the detection limit of the applied HPLC method and hence these have not been reported in the current work.

## 4. Discussion

Various available metabolic models and fluxes [8,27,28,29] as well as transcript and proteomic analyses [9,26] of different *P. thermoglucosidasius* strains have predicted or demonstrated the presence of genes and proteins involved in the complete metabolism of glucose to generate energy and biomass via both the TCA and glyoxylate cycles as well as through various fermentative pathways. Similarly, the basis of the WGS reaction in this bacterium has been demonstrated [10,14,25]. Here, the carboxydotroph *P. thermoglucosidasius* DSM 6285 was grown under different CO concentrations to further elucidate the mechanism of CO adaptation.

In contrast to strict anaerobic thermophile such as *Carboxydothermus hydrogenoformans* [30], the facultatively anaerobic *P. thermoglucosidasius* is incapable of using CO as sole carbon source. In our experiments, we observed that after the initial aerobic growth on glucose a subsequent fermentative rerouting of carbon fluxes occurred, and all cultures ultimately attained similar absorbance values. However, the OD_600_ of the cultures growing without CO continued to decline with the depletion of glucose and oxygen (Figure 4), whereas all cultures growing on CO showed a clear increase in absorbance regardless of CO concentration, indicating that the WGS reaction commenced (Figure 4b). In all instances, growth only ceases after the complete consumption of CO (Figure 1, Figure 2 and Figure 3). These observations suggest that, in addition to oxidizing CO to produce hydrogen [10,19], the WGS reaction in *P. thermoglucosidasius* is coupled to both energy conservation similar to the WGS reaction in *Rubrivivax gelatinosus* [16] and carbon assimilation into biomass. Further work is, however, necessary to determine the exact source and mechanism of carbon assimilation observed during the WGS reaction conditions.

Various life forms, including bacteria, show a wide range of physiological responses to CO. The gas either interferes with central metabolic pathways resulting in deprivation of energy and suppression of immune response or serves as sole or alternative source of energy and/or carbon [31]. *P. thermoglucosidasius* oxidises CO strictly under anaerobic conditions [10], which implies that during initial aerobic growth, the bacterium had to cope with the toxicity of CO to its terminal oxidases. 

Key reactions of the TCA/glyoxylate bypass generating reducing equivalents for oxidative phosphorylation in *P. thermoglucosidasius* include the reactions that produce oxoglutarate (NADPH), succinyl-CoA (NADH), fumarate (quinol) and oxaloacetate (quinol and NADH) catalysed by isocitrate dehydrogenase, 2-oxoglutarate dehydrogenase, succinate dehydrogenase and malate: quinone oxidoreductase/malate dehydrogenase, respectively (Figure 6). Electrons from reactions catalysed by the latter two membrane-bound enzymes [32,33] are donated to electron transfer chains in the form of quinones [34]. The observed increased production of acetate and lactate with increasing CO concentration (Figure 5) indicates a diversion of the reducing equivalents away from terminal oxidases. This suggests that the terminal oxidases in *P. thermoglucosidasius* are sensitive to CO, which, depending on the degree of stability of the complexes formed between gaseous substrates and respiratory oxidases [35,36], leads to a backlog of reducing equivalents. Instructively, the results indicate that the bacterium employs several strategies to cope with CO. Fermenting pyruvate to lactate immediately reduces the backlog of NADH, while the final step of acetate production catalysed by acetate kinase generates ATP that may offset the shortfall created by the effects of CO on the activity in the terminal oxidases. Previous studies have reported the redirection of carbon flux to accumulate acetate and lactate as a strategy used by *P. thermoglucosidasius* during transition from high to low redox conditions [8,9]. In addition, lactate production from pyruvate is a well-known strategy for rebalancing the pool of NAD^+^/NADH during bacterial growth on glucose [37].

In *Bacillus subtilis*, aerobic oxidation of lactate is catalysed by membrane bound iron-sulphur-containing lactate utilizing proteins (LutA-C) with oxygen serving as the terminal electron acceptor [38]. The draft genome of *P. thermoglucosidasius* harbours putative LutA-C protein complex genes, DV713_RS05720-30 (NZ_QQOK01000006.1: 86813-89694), which may serve as the pathway for the observed lactate utilization in the latter strain. Under CO-induced imbalance in redox potential, *P. thermoglucosidasius* therefore likely employs a system whereby NADH is oxidised to NAD^+^ via lactate production and the LutA-C complex subsequently catalyses the utilization of this lactate. Additionally, NADH is also oxidised via conversion of acetyl-CoA to ethanol, a reaction which is also discernible from the current results despite the trace amount of ethanol detected.

Similarly, acetate accumulation may provide an additional means of offsetting the redox imbalance [39,40] induced by CO and provides the organism with energy in the form of ATP [41,42]. In contrast to lactate, the recycling of acetate back to acetyl-CoA by acetyl-CoA synthetase (ACS) requires ATP [43,44], suggesting that the reassimilation of all acetate may result in zero net ATP but may provide an additional carbon source for growth. Expression of ACS encoding transcripts have been previously reported in *P. thermoglucosidasius* DSM 6285 over a relatively similar growth phase (microaerobic) in which acetate consumption was observed [26]. However, only a proportion of the acetate was channelled back into the central carbon metabolism owing to the requirement of energy for the reaction.

In the study presented here, no pyruvate and acetyl-CoA were quantified, which would have allowed direct conclusion about the distribution of metabolites between the TCA cycle and the glyoxylate shunt (GS). However, based on the observed production of glyoxylate and succinate, it can be assumed that a carbon flux via the GS occurred (Figure 6 and Figure 7a,b). Ferrying metabolites into the GS serves to prevent loss of carbon flux from 2-C compounds like acetyl-CoA by the CO_2_-producing steps of the TCA cycle and represents a major strategy when bacteria grow on 2-carbon substrates such as acetate [45,46]. Induction of GS has been reported in response to stresses, including oxidative [46,47,48,49] and antibiotic stress [50] in various microorganisms. The current data, however, suggest a negative effect of CO on the GS, evidenced by the decrease in glyoxylate concentration with increasing concentrations of CO. Strong suppression of GS has previously been reported under microaerobic conditions in *P. thermoglucosidasius* [9].

Whereas glyoxylate undergoes condensation with acetyl-CoA, as marked by the general decline in its concentration (Figure 6 and Figure 7a,b), succinate accumulates in both the presence and absence of CO (Figure 6 and Figure 7a,b). Accumulation of succinate in cultures grown without CO is surprising since previous proteomics studies reported the expression of the succinate dehydrogenase enzyme complex, which catalyses the conversion of succinate to fumarate in *P. thermoglucosidasius* [9]. As mentioned above, no extracellular fumarate was detected in the current study. Intracellular metabolites should therefore be considered in future studies. However, accumulation of succinate, especially in the CO-treated cultures, could be a strategy whereby the organism generates some ATP via the conversion of succinyl-CoA to succinate (Figure 6). Conceivably, succinate could have originated via both the GS and the complete TCA cycle depending on the CO levels. However, both the GS and the TCA cycle rely on the same key enzymes, isocitrate lyase and isocitrate dehydrogenase, and would therefore compete for the same substrate, isocitrate. The latter was reported to be upregulated at the protein level in *P. thermoglucosidasius* growing under microaerobic condition [9]. Nevertheless, succinate is an important substrate for several industrial applications [51] and therefore, these pathways may be optimized or engineered for its production.

## 5. Conclusions

In this study, we aimed to elucidate the effect of carbon monoxide on growth and carbon metabolism of *P. thermoglucosidasius* by exposing this carboxydotroph to atmospheres containing different concentrations of CO and tracing end products of the central carbon metabolism. The organism showed robust physiological versatility that allows it to circumvent the toxicity of CO on the terminal oxidases and the resultant perturbation of redox potential. *P. thermoglucosidasius* appears to cope by diverting the carbon flux through organic acids and other products which reduce the backlog of reducing equivalents and possibly generate energy. Additionally, the results indicate that the organism generates energy and potentially carbon via the WGS reaction. In summary, this study has further elucidated the metabolic mechanisms of *P. thermoglucosidasius*, which may enhance the development of strategies to improve the production of biohydrogen and other important by-products such as succinate, either by optimizing the redox balance or by metabolic engineering.

## Data Availability

Not applicable.

## References

[1] Aliyu H., Lebre P., Blom J., Cowan D., De Maayer P. (2016). Phylogenomic re-assessment of the thermophilic genus Geobacillus. Syst. Appl. Microbiol..

[2] Aliyu H., Lebre P., Blom J., Cowan D., De Maayer P. (2018). Corrigendum to “Phylogenomic re-assessment of the thermophilic genus Geobacillus”. Syst. Appl. Microbiol..

[3] Zeigler D.R. (2014). The *Geobacillus* paradox: Why is a thermophilic bacterial genus so prevalent on a mesophilic planet?. Microbiology.

[4] De Maayer P., Brumm P.J., Mead D.A., Cowan D.A. (2014). Comparative analysis of the *Geobacillus* hemicellulose utilization locus reveals a highly variable target for improved hemicellulolysis. BMC Genom..

[5] Brumm P., De Maayer P., Cowan D.A., MEAD D.A. (2015). Genomic analysis of six new *Geobacillus* strains reveals highly conserved carbohydrate degradation architectures and strategies. Front. Microbiol..

[6] Hussein A.H., Lisowska B.K., Leak D.J. (2015). The genus Geobacillus and their biotechnological potential. Advances in Applied Microbiology.

[7] Wada K., Suzuki H., Salwan R., Sharma V. (2020). Chapter 15—Biotechnological platforms of the moderate thermophiles, Geobacillus species: Notable properties and genetic tools. Physiological and Biotechnological Aspects of Extremophiles.

[8] Tang Y.J., Sapra R., Joyner D., Hazen T.C., Myers S., Reichmuth D., Blanch H., Keasling J.D. (2009). Analysis of metabolic pathways and fluxes in a newly discovered thermophilic and ethanol-tolerant Geobacillus strain. Biotechnol. Bioeng..

[9] Loftie-Eaton W., Taylor M., Horne K., Tuffin M.I., Burton S.G., Cowan D.A. (2013). Balancing redox cofactor generation and ATP synthesis: Key microaerobic responses in thermophilic fermentations. Biotechnol. Bioeng..

[10] Mohr T., Aliyu H., Küchlin R., Polliack S., Zwick M., Neumann A., Cowan D., De Maayer P. (2018). CO-dependent hydrogen production by the facultative anaerobe Parageobacillus thermoglucosidasius. Microb. Cell Factories.

[11] Antonopoulou G., Ntaikou I., Stamatelatou K., Lyberatos G. (2011). Biological and fermentative production of hydrogen. Handbook of Biofuels Production.

[12] Henstra A.M., Sipma J., Rinzema A., Stams A.J.M. (2007). Microbiology of synthesis gas fermentation for biofuel production. Curr. Opin. Biotechnol..

[13] Schoelmerich M.C., Müller V. (2019). Energy conservation by a hydrogenase-dependent chemiosmotic mechanism in an ancient metabolic pathway. Proc. Natl. Acad. Sci. USA.

[14] Adachi Y., Inoue M., Yoshida T., Sako Y. (2020). Genetic Engineering of Carbon Monoxide-dependent Hydrogen-producing Machinery in Parageobacillus thermoglucosidasius. Microbes Environ..

[15] Søndergaard D., Pedersen C.N.S., Greening C. (2016). HydDB: A web tool for hydrogenase classification and analysis. Sci. Rep. UK.

[16] Maness P.-C., Huang J., Smolinski S., Tek V., Vanzin G. (2005). Energy generation from the CO oxidation-hydrogen production pathway in Rubrivivax gelatinosus. Appl. Environ. Microbiol..

[17] Lim J.K., Mayer F., Kang S.G., Müller V. (2014). Energy conservation by oxidation of formate to carbon dioxide and hydrogen via a sodium ion current in a hyperthermophilic archaeon. Proc. Natl. Acad. Sci. USA.

[18] Sapra R., Bagramyan K., Adams M.W.W. (2003). A simple energy-conserving system: Proton reduction coupled to proton translocation. Proc. Natl. Acad. Sci. USA.

[19] Fukuyama Y., Inoue M., Omae K., Yoshida T., Sako Y., Gadd G.M., Sariaslani S. (2020). Chapter Three—Anaerobic and hydrogenogenic carbon monoxide-oxidizing prokaryotes: Versatile microbial conversion of a toxic gas into an available energy. Advances in Applied Microbiology.

[20] Robb F.T., Techtmann S.M. (2018). Life on the fringe: Microbial adaptation to growth on carbon monoxide. F1000Research.

[21] Nakagawa T., Iino T., Suzuki K.-i., Harayama S. (2006). Ferrimonas futtsuensis sp. nov. and Ferrimonas kyonanensis sp. nov., selenate-reducing bacteria belonging to the Gammaproteobacteria isolated from Tokyo Bay. Int. J. Syst. Evol. Microbiol..

[22] Srinivas T.N.R., Vijaya Bhaskar Y., Bhumika V., Anil Kumar P. (2013). Photobacterium marinum sp. nov., a marine bacterium isolated from a sediment sample from Palk Bay, India. Syst. Appl. Microbiol..

[23] Omae K., Fukuyama Y., Yasuda H., Mise K., Yoshida T., Sako Y. (2019). Diversity and distribution of thermophilic hydrogenogenic carboxydotrophs revealed by microbial community analysis in sediments from multiple hydrothermal environments in Japan. Arch. Microbiol..

[24] Mohr T., Aliyu H., Küchlin R., Zwick M., Cowan D., Neumann A., de Maayer P. (2018). Comparative genomic analysis of Parageobacillus thermoglucosidasius strains with distinct hydrogenogenic capacities. BMC Genom..

[25] Mohr T., Aliyu H., Biebinger L., Gödert R., Hornberger A., Cowan D., de Maayer P., Neumann A. (2019). Effects of different operating parameters on hydrogen production by Parageobacillus thermoglucosidasius DSM 6285. AMB Express.

[26] Aliyu H., Mohr T., Cowan D., de Maayer P., Neumann A. (2020). Time-course Transcriptome of Parageobacillus thermoglucosidasius DSM 6285 Grown in the Presence of Carbon Monoxide and Air. Int. J. Mol. Sci..

[27] Mol V., Bennett M., Sánchez B.J., Lisowska B.K., Herrgård M.J., Nielsen A.T., Leak D.J., Sonnenschein N. (2021). Genome-scale metabolic modelling of *P. thermoglucosidasius* NCIMB 11955 reveals metabolic bottlenecks in anaerobic metabolism. bioRxiv.

[28] Karp P.D., Paley S., Romero P. (2002). The Pathway Tools software. Bioinformatics.

[29] Ahmad A., Hartman H.B., Krishnakumar S., Fell D.A., Poolman M.G., Srivastava S. (2017). A Genome Scale Model of Geobacillus thermoglucosidasius (C56-YS93) reveals its biotechnological potential on rice straw hydrolysate. J. Biotechnol..

[30] Henstra A.M., Stams A.J.M. (2004). Novel Physiological Features of *Carboxydothermus hydrogenoformans* and *Thermoterrabacterium ferrireducens*. Appl. Environ. Microbiol..

[31] Hopper C.P., De La Cruz L.K., Lyles K.V., Wareham L.K., Gilbert J.A., Eichenbaum Z., Magierowski M., Poole R.K., Wollborn J., Wang B. (2020). Role of Carbon Monoxide in Host–Gut Microbiome Communication. Chem. Rev..

[32] Molenaar D., van der Rest M.E., Petrović S. (1998). Biochemical and genetic characterization of the membrane-associated malate dehydrogenase (acceptor) from Corynebacterium glutamicum. Eur. J. Biochem..

[33] Cecchini G. (2003). Function and structure of complex II of the respiratory chain. Annu. Rev. Biochem..

[34] van der Rest M.E., Frank C., Molenaar D. (2000). Functions of the Membrane-Associated and Cytoplasmic Malate Dehydrogenases in the Citric Acid Cycle of *Escherichia coli*. J. Bacteriol..

[35] Borisov V.B., Forte E., Sarti P., Brunori M., Konstantinov A.A., Giuffrè A. (2007). Redox control of fast ligand dissociation from Escherichia coli cytochrome bd. Biochem. Biophys. Res. Commun..

[36] Giuffrè A., Borisov V.B., Arese M., Sarti P., Forte E. (2014). Cytochrome bd oxidase and bacterial tolerance to oxidative and nitrosative stress. Biochim. Et Biophys. Acta Bioenerg..

[37] Clark D.P. (1989). The fermentation pathways of Escherichia coli. Fems Microbiol. Rev..

[38] Chai Y., Kolter R., Losick R. (2009). A Widely Conserved Gene Cluster Required for Lactate Utilization in *Bacillus subtilis* and Its Involvement in Biofilm Formation. J. Bacteriol..

[39] Cypionka H., Meyer O. (1983). Carbon monoxide-insensitive respiratory chain of Pseudomonas carboxydovorans. J. Bacteriol..

[40] Wareham L.K., Begg R., Jesse H.E., Van Beilen J.W.A., Ali S., Svistunenko D., McLean S., Hellingwerf K.J., Sanguinetti G., Poole R.K. (2016). Carbon Monoxide Gas Is Not Inert, but Global, in Its Consequences for Bacterial Gene Expression, Iron Acquisition, and Antibiotic Resistance. Antioxid. Redox Signal..

[41] Hasona A., Kim Y., Healy F.G., Ingram L.O., Shanmugam K.T. (2004). Pyruvate Formate Lyase and Acetate Kinase Are Essential for Anaerobic Growth of *Escherichia coli* on Xylose. J. Bacteriol..

[42] Klein A.H., Shulla A., Reimann S.A., Keating D.H., Wolfe A.J. (2007). The Intracellular Concentration of Acetyl Phosphate in *Escherichia coli* Is Sufficient for Direct Phosphorylation of Two-Component Response Regulators. J. Bacteriol..

[43] Pinhal S., Ropers D., Geiselmann J., de Jong H. (2019). Acetate Metabolism and the Inhibition of Bacterial Growth by Acetate. J. Bacteriol..

[44] Wolfe A.J. (2005). The Acetate Switch. Microbiol. Mol. Biol. Rev..

[45] Shinar G., Rabinowitz J.D., Alon U. (2009). Robustness in Glyoxylate Bypass Regulation. PLoS Comput. Biol..

[46] Ahn S., Jung J., Jang I.-A., Madsen E.L., Park W. (2017). Role of Glyoxylate Shunt in Oxidative Stress Response. J. Biol. Chem..

[47] Rui B., Shen T., Zhou H., Liu J., Chen J., Pan X., Liu H., Wu J., Zheng H., Shi Y. (2010). A systematic investigation of Escherichia coli central carbon metabolism in response to superoxide stress. BMC Syst. Biol..

[48] Li K., Pidatala R.R., Ramakrishna W. (2012). Mutational, proteomic and metabolomic analysis of a plant growth promoting copper-resistant Pseudomonas spp. FEMS Microbiol. Lett..

[49] Schroeter R., Voigt B., Jürgen B., Methling K., Pöther D.C., Schäfer H., Albrecht D., Mostertz J., Mäder U., Evers S. (2011). The peroxide stress response of Bacillus licheniformis. Proteomics.

[50] Nandakumar M., Nathan C., Rhee K.Y. (2014). Isocitrate lyase mediates broad antibiotic tolerance in Mycobacterium tuberculosis. Nat. Commun..

[51] Durall C., Kukil K., Hawkes J.A., Albergati A., Lindblad P., Lindberg P. (2021). Production of succinate by engineered strains of Synechocystis PCC 6803 overexpressing phosphoenolpyruvate carboxylase and a glyoxylate shunt. Microb. Cell. Fact..

